# 2790. Activity of Cefiderocol and Comparator Agents Against Pediatric Isolates of Enterobacterales, *Pseudomonas aeruginosa*, *Acinetobacter baumannii-calcoaceticus* species complex, and *Stenotrophomonas maltophilia* from the SENTRY Surveillance Program (2020-2022)

**DOI:** 10.1093/ofid/ofad500.2401

**Published:** 2023-11-27

**Authors:** Sean T Nguyen, Boudewijn L DeJonge, Jason J Bryowsky, Anne Henriksen, Christopher M Longshaw, Joshua Maher, Rodrigo E Mendes, Miki Takemura, Yoshinori Yamano

**Affiliations:** Shionogi Inc., Florham Park, New Jersey; Shionogi Inc., Florham Park, New Jersey; Shionogi Inc., Florham Park, New Jersey; Shionogi BV, Copenhagen, Hovedstaden, Denmark; Shionogi B.V., London, England, United Kingdom; JMI Laboratories, North Liberty, Iowa; JMI Laboratories, North Liberty, Iowa; Shionogi & Co., Ltd, Toyonaka, Osaka, Japan; Shionogi & Co., Ltd., Toyonaka, Osaka, Japan

## Abstract

**Background:**

Cefiderocol (CFDC) is a siderophore cephalosporin with broad activity against Gram-negative bacteria including multi-drug resistant isolates. The *in vitro* activity of CFDC and comparator agents was evaluated against pediatric (0-17 years old) isolates collected in 2020–2022 as part of the SENTRY Antimicrobial Surveillance Program.

In vitro Activity of Cefiderocol and Comparator Agents Against Pediatric Isolates

**Figure:**
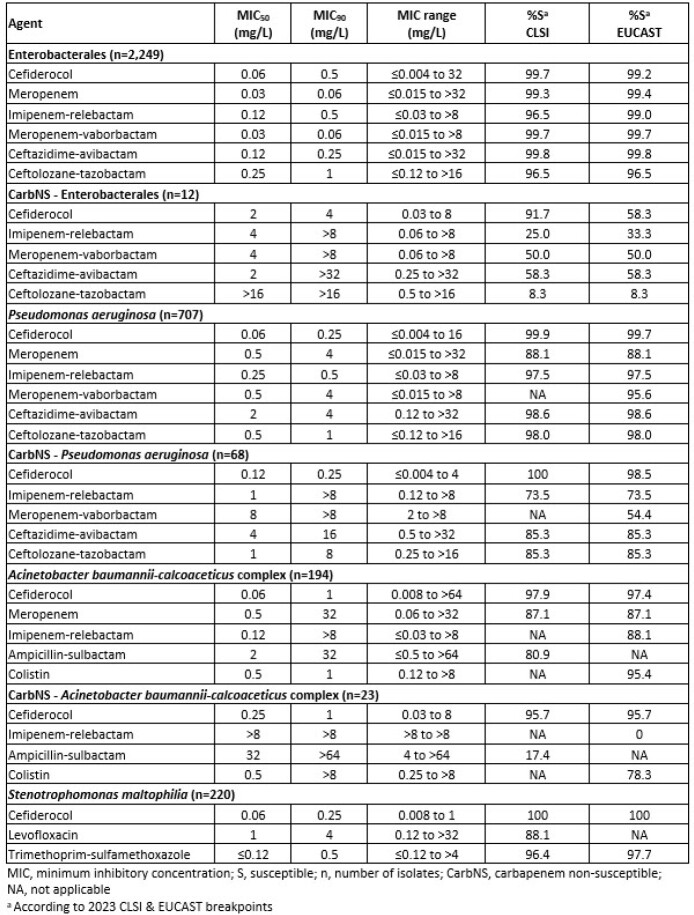

**Methods:**

2,249 Enterobacterales (ENT), 707 *P. aeruginosa*, 194 *Acinetobacter baumannii-calcoaceticus* complex (ABC) and 220 *S. maltophilia* from the USA and Europe were tested for susceptibility (%S) by broth microdilution with cation-adjusted Mueller-Hinton broth (CAMHB). Iron-depleted CAMHB was applied for CFDC. Comparators included newer β-lactam/β-lactamase inhibitor (BL/BLI) combinations ceftazidime-avibactam (CZA), ceftolozane-tazobactam (C/T), imipenem-relebactam (I-R) and meropenem-vaborbactam (MVB) as well as ampicillin/sulbactam (SAM) meropenem (MEM) and colistin (CST). %S was interpreted according to 2023 CLSI & EUCAST breakpoints. Carbapenem non-susceptible (CarbNS) was defined as non-susceptibility to imipenem and MEM.

**Results:**

All agents displayed >96 %S for ENT while CFDC (MIC_50/90_, 2/4 mg/L; 91.7 %S) was the most active agent against CarbNS ENT. *P. aeruginosa* susceptibilities to CFDC and BL/BLI combinations were >97.0%. CFDC was the most potent agent against CarbNS *P. aeruginosa* with MIC_50/90_ values of 0.12/0.25 mg/L and 100 %S & 98.5 %S per CLSI & EUCAST breakpoints, respectively. ABC susceptibility to CFDC was >97% per CLSI & EUCAST while the susceptibility for MEM was 87.1% (CLSI & EUCAST) and SAM was 80.9% (CLSI). CFDC (MIC_50/90_, 0.25/1 mg/L; 95.7 %S) displayed better *in vitro* potency in CarbNS ABC as compared to SAM (MIC_50/90_, 32/ >64 mg/L; 17.4 %S CLSI) and CST (MIC_50/90_, 0.5/ >8 mg/L; 78.3%S EUCAST). Among pediatric *S. maltophilia*, CFDC was the most active agent with MIC_50/90,_ 0.06/0.25 and 100 %S per CLSI & EUCAST breakpoints.

**Conclusion:**

CFDC was a highly active β-lactam against contemporary pediatric isolates of Enterobacterales, *P. aeruginosa*, ABC, and *S. maltophilia*, including CarbNS subsets for which treatment options are limited. These data suggest CFDC may be a valuable treatment for serious Gram-negative infections in pediatric patients.

**Disclosures:**

**Sean T. Nguyen, PharmD**, Shionogi: Employee|Shionogi, Inc: Employee **Boudewijn L. DeJonge, PhD**, Shionogi Inc.: Employee **Jason J. Bryowsky, PharmD, MS**, Shionogi Inc.: Employee **Anne Henriksen, PhD**, Shionogi: Employee **Christopher M. Longshaw, PhD**, Shionogi BV: Employee **Joshua Maher, PhD**, AbbVie: Grant/Research Support|Affinity Biosensors: Grant/Research Support|AimMax Therapeutics, Inc: Grant/Research Support|Alterity Therapeutics: Grant/Research Support|Amicrobe, Inc: Grant/Research Support|Arietis Pharma: Grant/Research Support|Armata Pharmaceuticals, Inc: Grant/Research Support|Astrellas Pharma, Inc.: Grant/Research Support|Basilea Pharmaceutica AG: Grant/Research Support|Becton Dickinson And Company: Grant/Research Support|bioMerieux, Inc: Grant/Research Support|Boost Biomes: Grant/Research Support|Diamond V: Grant/Research Support|Fedora Pharmaceuticals, Inc: Grant/Research Support|Iterum Therapeutics plc: Grant/Research Support|Johnson & Johnson: Grant/Research Support|Kaleido Biosciences, Inc.: Grant/Research Support|Meiji Seika Pharma Co. Ltd.: Grant/Research Support|National Institutes of Health: Grant/Research Support|Pfizer Inc.: Grant/Research Support|Roche Holding AG: Grant/Research Support|Shionogi Inc.: Grant/Research Support|Summmit Therapeutics, Inc.: Grant/Research Support|Zoetis Inc: Grant/Research Support **Rodrigo E. Mendes, PhD**, AbbVie: Grant/Research Support|Basilea: Grant/Research Support|Cipla: Grant/Research Support|Entasis: Grant/Research Support|GSK: Grant/Research Support|Paratek: Grant/Research Support|Pfizer: Grant/Research Support|Shionogi: Grant/Research Support **Miki Takemura, n/a**, Shionogi & Co., Ltd.: Stocks/Bonds **Yoshinori Yamano, PhD**, Shionogi HQ: Employee

